# Gender Differences in Social Isolation and Loneliness Among a National Sample of Dementia Caregivers

**DOI:** 10.1093/geroni/igaf122.732

**Published:** 2025-12-31

**Authors:** Mary Louise Pomeroy, Yiqing Qian, Gilbert Gimm, Katherine Ornstein, Thomas Cudjoe

**Affiliations:** Johns Hopkins Bloomberg School of Public Health, Baltimore, Maryland, United States; Johns Hopkins University School of Nursing, Baltimore, Maryland, United States; George Mason University, Fairfax, Virginia, United States; Johns Hopkins University, Baltimore, Maryland, United States; Johns Hopkins School of Medicine, Baltimore, Maryland, United States

## Abstract

As life expectancy rises in the U.S., more older adults and their family/friend caregivers will face the challenges of managing dementia. Social connections can play a crucial role in providing support that may attenuate these challenges. However, little is known about gender differences in social isolation and loneliness among dementia caregivers. This study used Round 12 of the National Study of Caregiving (NSOC) to examine social isolation and loneliness among a nationally-representative sample of caregivers to community-dwelling older adults with dementia in 2022. Among 753 dementia caregivers, 39% (n = 242) were men. Using chi-squared tests and a multi-domain social isolation typology, we found that male caregivers were more likely to be socially isolated compared to female caregivers (21.4% vs. 12.4%; p < 0.01). Underlying components driving isolation among men included small core discussion network size (25.4% vs. 15.4%; p < 0.05) and no religious service attendance (64.6% vs. 45.5%; p < 0.001). However, men were less likely to live alone (3.9% vs. 12.6%; p < 0.001) and there were no significant gender differences in participation in community-based activities (66.5% vs. 64.5%, p = 0.74). Male caregivers were less likely to report loneliness than females, though this difference was not significant (23.8% vs. 30.0%, p = 0.12). Because women are at greater risk for dementia than men, the number of male caregivers for women with dementia is expected to grow. However, male caregivers are underrepresented in dementia research. Interventions and policies to support dementia care management (e.g., the GUIDE model) should carefully consider gender differences in social connection among dementia caregivers.

